# Neonatal Features of the Prader-Willi Syndrome; The Case for Making the Diagnosis During the First Week of Life

**DOI:** 10.4274/jcrpe.0029

**Published:** 2018-07-31

**Authors:** Filiz Mine Çizmecioğlu, Jeremy Huw Jones, Wendy Forsyth Paterson, Sakina Kherra, Mariam Kourime, Ruth McGowan, M. Guftar Shaikh, Malcolm Donaldson

**Affiliations:** 1 Kocaeli University Faculty of Medicine, Department of Paediatric Endocrinology and Diabetes, Kocaeli, Turkey; 2 Royal Hospital for Children, Clinic of Endocrinology, Glasgow, United Kingdom; 3 CHU Parnet, Clinic of Pediatrics, Algiers, Algeria; 4 University Hospital Abderrahim Harouchi, Casablanca, Morocco; 5 Southern Glasgow University Hospital, West of Scotland Genetic Services, Glasgow, United Kingdom; 6 University of Glasgow Faculty of Medicine, Royal Hospital for Children, Clinic of Child Health, Glasgow, United Kingdom

**Keywords:** Prader-Willi syndrome, hypotonia, fetal movement, nasogastric feeding

## Abstract

**Objective:**

Early diagnosis is of proven benefit in Prader-Willi syndrome (PWS). We therefore examined key perinatal features to aid early recognition.

**Methods:**

Data were collected from case records of subjects attending a multi-disciplinary clinic and from a retrospective birth questionnaire.

**Results:**

Ninety patients (54 male-36 female) were seen between 1991-2015, most with paternal deletion (n=56) or maternal isodisomy (n=26). Features included cryptorchidism in 94% males, preterm birth (26%), birthweight <2500 g (24%), polyhydramnios (23%), breech presentation (23%) and need for nasogastric feeding (83%). Reduced fetal movements (FM) were reported in 82.5% patients compared with 4% healthy siblings. Of 35 children born since 1999, 23 were diagnosed clinically within 28 days while diagnosis in 12 was >28 days: 1-12 months in seven; and 3.75-10.5 years in five. Typical PWS features in these 12 infants included hypotonia (100%), feeding difficulties (75%), cryptorchidism (83% males) and reduced FM (66%). Causes other than PWS including neuromuscular disease were considered in nine patients.

**Conclusion:**

Neonatal hypotonia, reduced FM, feeding difficulties and cryptorchidism should immediately suggest PWS, yet late diagnosis continues in some cases. Awareness of the typical features of PWS in newborn units is required to allow prompt detection even in the presence of confounding factors such as prematurity.

## What is already known on this topic?

Early diagnosis in Prader-Willi syndrome allows appropriate management of feeding difficulties to be implemented and is important for counselling as well as minimising parental uncertainty and anxiety. The prevalence of preterm birth and low birthweight is increased in Prader-Willi syndrome and is a potential source of diagnostic confusion.

## What this study adds?

Although the prevalence of preterm birth (26%) and low birthweight (24%) is increased in Prader-Willi syndrome, diagnosis in preterm infants is not significantly delayed. However, clinical diagnosis in our cohort was made later than 28 days in 34% of the patients despite presence of classic features of hypotonia and cryptorchidism in males and need for nasogastric feeding in over 80%. Comparison with non-affected siblings shows that mothers detect reduced fetal movement in 80% of patients with Prader-Willi syndrome, a feature which can facilitate early diagnosis. 

## Introduction

Prader-Willi syndrome (PWS) is a neurodevelopmental disorder resulting from absence of paternally imprinted genes in the 15q11-13 region, caused by deletion in the paternal chromosome (del15q) in about 70% of cases or disomy of the maternal chromosome [maternal uniparental disomy of chromosome 15 (mUPD15)] in about 25% (1). The natural history of PWS is complex, with different phenotypic features developing at different ages (2,3).Before the advent of confirmatory molecular genetic testing, diagnosis was made solely according to age-related clinical criteria (4).Prompt diagnosis of PWS is not only beneficial for educating families about the condition but also for facilitating early therapeutic interventions such as speech therapy, physiotherapy and dietetics to help with feeding difficulties and prevent early onset obesity (5,6,7). Moreover, timely diagnosis will enable growth hormone (GH) therapy to be implemented early so as to improve linear growth and motor development (8). So typical are the features of PWS that, especially with publications highlighting the phenotype (9,10,11,12,13,14) and widely available molecular genetic analysis, it might be expected that diagnosis would be made at, or shortly after, birth.

Hypotonia is a relatively common problem in the newborn period, affecting not only otherwise healthy preterm infants but also those with sepsis, neurological disease and metabolic disorders. The challenge to the clinician, therefore, is to identify the minority of infants with PWS from the majority of babies in whom hypotonia is a transient feature and from those who are suffering from a different condition such as spinal muscular atrophy (SMA) (15). Our clinical experience indicates that the diagnosis of PWS continues to be made beyond the newborn period in some cases.

An increased incidence of preterm birth and low birth weight (LBW) has been reported in PWS, together with obstetric symptoms such as reduced fetal movement (FM), polyhydramnios and malpresentation (16,17,18). Some typical features of PWS infants, such as hypotonia and inability to suck necessitating tube feeding, are also common in unaffected preterm infants. It might be conjectured, therefore, that the diagnosis of PWS is more difficult when the infant is preterm.

A multidisciplinary PWS clinic has been held at our centre in the Royal Hospital for Children in Glasgow since 1991, providing data on a heterogeneous Scottish cohort. The aim of the study was to identify key diagnostic features of PWS to facilitate early diagnosis. These include obstetric and newborn data, feeding difficulties requiring assisted feeding and the prevalence of reduced FMs. In addition, we looked for an association between prenatal and perinatal characteristics and genotype, to further develop previous work (16,17,18).

## Methods

All patients with proven PWS attending the multidisciplinary clinic in Glasgow, Scotland, since its inception in 1991 until December 2015 were included. Data on genotype, BW, gestation, delivery method, obstetric symptoms, postnatal complications and timing of diagnosis were collected and analysed from three sources: a birth questionnaire completed by the parent(s); the subject’s case records; and maternal obstetric records, when available. It was decided, however, that the systematic recording of phenotypic features such as hair and eye colour, bifrontal narrowing, characteristic eyes, small hands and feet and weak cry should not be included within the scope of the study. This is because these data were recorded inconsistently, if at all, during the newborn period and by multiple observers and it was felt appropriate to confine this retrospective study to the collection of hard data.

Endocrine problems were documented by detailing the number of patients undergoing GH stimulation testing either with insulin or arginine and the outcome, and defining GH deficiency as peak values <15 mU/L. The numbers of patients treated with GH injections as well as the numbers of patients diagnosed with either hypothyroidism or cortisol deficiency were also documented.

The questionnaire invited parents with more than one child to submit information on the BW, gestation and delivery method of their other children. Mothers were also asked to retrospectively estimate the degree of* in utero* FM in their affected and unaffected children using a simple 5 point scoring system ranging from 1: much less than expected to 5: much more than expected.

BW was assessed according to United Kingdom reference standards (Least Mean Square Growth program; http://www.healthforallchildren.com) and expressed in kilograms and standard deviations (SD). The incidences of preterm birth (gestation <37 completed weeks), LBW, defined as BW <2500 g, small for gestational age (SGA), defined as BW <10^th^ percentile and operative delivery were compared with healthy siblings and contemporary Scottish population data (19).

### Statistical Analysis

All analyses were done using Minitab (version 13.1) at a significance level of 5%. Data distribution was assessed for normality using Anderson-Darling test. Parametric data are presented as mean (± SD) and non-parametric data as median (range) or median (range) (interquartile range) Quantitative variables were compared using t-tests and analysis of variance or Kruskal-Wallis and Mann-Whitney U tests. Qualitative variables were compared using chi-squared or Fisher’s exact tests.

### Ethical Aspects

Approval was initially granted by the Ethical Committee of the Royal Hospital for Sick Children in Glasgow in 2004 and data collection completed in 2011. Following the tragic loss of the lead investigator, Wendy Paterson in 2012, the study was relaunched in 2015 to update the data and to include more recent patients. This second phase of the study was approved by the National Health Service of the United Kingdom Research Ethics Authority (reference 15/NW/0900). In both study periods written informed consent was obtained from parents/guardians and subjects aged ≥16 years while children <16 years were invited to give assent.

## Results

Ninety (54 male) subjects with PWS born between 1950 and 2015 (median 1994) were included in the study (Table 1). Excluding eight deaths (male/female=5/3) at the ages of 15, 18, 23, 25, 27, 27, 29 and 45 years, the median (range) age of 82 patients on 01/01/2016 was 20.8 (0.9-65.7) years. Twenty-nine patients had undergone GH stimulation testing of whom 17 were GH insufficient-median (range) levels 7.2 (<0.1-14.8) mU/L; and 12 were GH sufficient-levels 31.6 (17.4-78.1 mU/L). A total of 20 patients were treated with GH injections during the study period. Hypothyroidism was not documented, and no patient was referred with elevation of thyroid stimulating hormone on newborn screening programme, which was introduced in Scotland in 1979. No patient was diagnosed with adrenal insufficiency during the study period, or received hydrocortisone therapy.

Questionnaires were sent to 72 families and returned by 60 (83%). Reasons for questionnaires not being sent to 18 families included adoption/fostering and parents no longer being contactable. Comparative birth data were collected for 97 siblings (56 male).

### Genetics

Genetic diagnosis of PWS was confirmed in 89 of the 90 subjects, as follows: paternal deletion (del15q) in 56 (62%); maternal disomy (mUPD15) in 26 (29%); translocation (chromosome 15-16) in one (1%); imprinting defect in two (2%) and four tested elsewhere (4%). One subject was initially reported to have no defect on standard PWS genetic screening but later Comparative Genomic Hybridization Array analysis revealed a deletion in paternal 15q11.2 incorporating the *NDN* (NECDIN) gene (OMIM 602117). *NDN* is important for normal hypothalamic development and it has been suggested that loss of *NDN* contributes to both the hypothalamic hypogonadism and neurological features of PWS (19). No defect was found in one other, older subject who nevertheless satisfied the Holm consensus criteriafor PWS (4).

### Prenatal, Perinatal and Postnatal Data

Data for all PWS subjects and a comparison between term and preterm infants are shown in Table 1. Mean (± SD) BW was significantly lower in the study group versus healthy siblings: 2.68 (0.6) (n=89) vs 3.34 (0.6) kg (n=91); and -1.03 (1.03) vs -0.182 (1.02) SD (p<0.001); prevalence of SGA significantly higher -32/89 (36%) versus 10/91 (11%). Median (range) PWS gestation was lower at 39 (30-43) versus 40 (33-42) weeks while preterm birth occurred significantly more often; in 23 (25.8%) patients compared with three (3.2%) siblings (p=0.03). 

Polyhydramnios was reported in 10/44 (22.7%) mothers. Breech presentation was recorded in 15/65 (23.1%) with one transverse lie. Mode of delivery was spontaneous vaginal in 38/86 subjects (44%), Caesarean section in 38/86 (44%), forceps-assisted delivery in six and ventouse extraction in three. FMs were reduced with a median score in PWS of 1 in the preterm and 2 in the term infants respectively (Table 1). 

**Figure 1:**
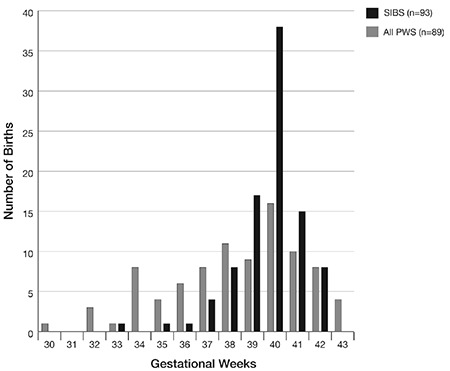
Comparison of gestational age between 89 Prader-Willi syndrome patients and 93 unaffected siblings Fetal movements were retrospectively scored by the mothers as 1: much less than expected, 2: a bit less than expected, 3: about as much as expected, 4: a bit more than expected and 5: a lot more than expected Sibs: siblings, PWS: Prader-Willi syndrome

Figure 1 shows that whereas sibling gestation was normally distributed, the PWS group suggested a bimodal, flattened distribution with peaks at both 34 and 40 weeks. This was more marked in the del15q than the mUPD15 groups.

### Fetal Movement Scores

**Figure 2:**
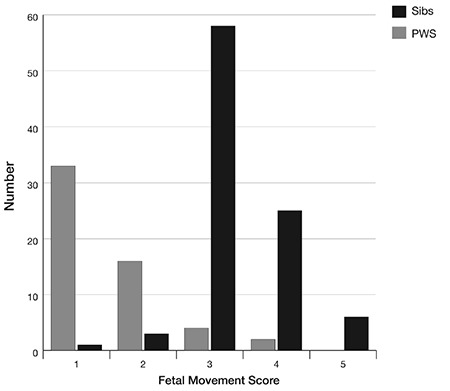
Comparison of fetal movement in 55 Prader-Willi syndrome subjects and 93 unaffected siblings Sibs: siblings, PWS: Prader-Willi syndrome

Decreased FM were present in 66/80 (82.5%) with normal movement in 14 (17.5%). Figure 2 shows comparison between 55 patients (including six with normal movement) and their unaffected siblings, with significantly lower scores for PWS (p<0.001). Mothers who had had non-affected babies (n=55) scored their affected babies significantly lower (p=0.02) than those who had had only one (affected) baby (n=25)-median scores 1 versus 2. Thus 8/25 (32%) of the mothers with experience of only one pregnancy, carrying a PWS-affected infant, reported normal FMs compared with 6/55 (11%) of those with more than one child although the FM scores for the former group were still significantly lower than for unaffected children (p<0.001).

### Feeding Difficulties

Nasogastric feeding (NGF) was given to 75 (86%) infants with no assisted feeding in 12 and no data in three. Six of the infants who did not receive assisted feeding were diagnosed after four months of age. Median (range) duration of NGF in days was 30 (2-480) (n=71) and was longer in preterm (n=21) at 42 (2-231) than in term infants (n=49) at 25 (3-480).

### Hypogonadism in Male Prader-Willi Syndrome Infants

Data on testicular position was available in all but five of the 54 males. Forty-six (94%) of the 49 boys had cryptorchidism, which was bilateral in 40, unilateral in five, unspecified in one. Only three (5.5%) male patients had normally descended testes. Cryptorchidism was present in all but one of the 12 preterm males.

### Hypoplasia of Labia Minora in Female Prader-Willi Syndrome Infants

This was documented as present in six girls at birth, not present (i.e. normal) in a further six, and not documented otherwise.

### Timing of Clinical and Molecular Diagnosis

Table 1 shows that age at clinical diagnosis was extremely variable during the study period and also that preterm infants were diagnosed sooner than term infants although this difference was not significant. 

Table 2 shows data on the timing of clinical (n=87) and genetic diagnosis (n=83) with subjects stratified by decade of birth. Median (range) age at diagnosis during the study period was 2.3 months (1 day-46 years) for clinical and 10 months (4 days-46.5 years) for genetic diagnosis. Since 1991, time to genetic diagnosis has been 28 days (4 days-10.6 years). Age at diagnosis showed an apparent improvement during the study period although the number of as yet undiagnosed patients, particularly during the 2010-2015 period, remains uncertain.

### Age at Clinical Diagnosis in Patients Born from the Year 2000 Onwards

Analysis of patients born since 1 January 2000 showed that of 35 patients (16 males) the clinical diagnosis was made within the first week of life in 12 infants, between days 8 and 27 in 11, aged 4 weeks to 12 months in seven, and after 5 years in five. Among the 12 infants who were diagnosed clinically within a week of birth, sepsis was considered in two and neuromuscular disease in another. Table 3 gives details on the 12 patients diagnosed ≥4 weeks from year 2000 onwards. Feeding difficulties and hypotonia at birth were present in all these patients, with NGFs given to nine. Other features included reduced FMs (9), prematurity (4), LBW (4), and bilateral cryptorchidism (5/6 males). Hypotonia was attributed in three cases to birth asphyxia, birth injury and benign congenital hypotonia. Magnetic resonance imaging (MRI) was carried out in three. Five children had developed hyperphagic obesity by the time that PWS was confirmed.

### Genotype vs Prenatal and Perinatal Features

Table 4 shows that both maternal and paternal age were significantly greater in the mUPD15 group compared with the del15q group. Also, median year of birth was later in the mUPD15 group than in the del15q group-2004 vs 1994-although this was not significant (p=0.11). The prevalence of diminished FMs, birthweight and gestation and operative delivery was similar between the two groups.

## Discussion

This study of 90 patients with PWS, drawn from a geographically defined area over a 25-year period, confirms and extends existing knowledge of the prenatal, perinatal and postnatal features of PWS, the dominant trait being presence of hypotonia. It should be noted that our study is confined to describing only those infants whose hypotonia was caused by PWS so that the prevalence of neonatal hypotonia in Scotland during the study period and hence the proportion of those hypotonic infants who actually had PWS is unknown.

Intrauterine hypotonia causes polyhydramnios, malpresentation and reduced FMs leading to an increased prevalence of operative delivery. All these features were seen in this study, consistent with previous reports (9,12,17,18). Of note, breech presentation was found in 23% of our cohort compared with 0.2% in the Scottish population while 44% were delivered by caesarean section in contrast to Scottish population data showing a rate of 8.6% in 1975-76, rising to 29.9% in 2014-15 (20).

We found increased incidence of LBW (23.6%) and SGA (36%), contrasting with 12.7% for SGA in the Scottish population (21) although SGA prevalence in PWS has been reported to be higher, ranging from 53-65% (9,22). It has been suggested that SGA in PWS may result from a failure to express paternal genes which improve placental function (22).

The prevalence of preterm birth (25.8%) in our cohort markedly exceeds the current Scottish figures of 7.3% (19). This increase is similar to Lionti’s study (27.4%) (22) and higher than two earlier French studies which reported rates of 13/86 (15%) and 2/19 (10.5%) versus 6% in the general population (12,17).Preterm birth in PWS may be partly due to polyhydramnios from reduced fetal swallowing causing uterine distension and inducing early labour (14,17). The distribution of gestational age seen in our cohort appeared to be bimodal. This finding has been previously reported by Butler et al (16). However these authors report bimodal distribution only in mUPD15 whereas we found bimodality only in the deletion group. More data on larger populations are needed to confirm this possible bimodal pattern.

In contrast to our expectations that features in common with prematurity might cause delay in the diagnosis of PWS, we found that affected premature infants tended to be diagnosed earlier than their term counterparts. This might be attributable to preterm infants receiving more clinical attention as well as having a longer stay in hospital.

While most prenatal features of PWS, such as polyhydramnios and abnormal presentation are too non-specific to be of value, reduced FM is of importance in indicating the congenital nature of the hypotonia, prompting consideration of disorders such as PWS or neurological conditions such as SMA. Careful comparison of maternal retrospective evaluation of FMs in affected and unaffected infants shows a striking difference, with 82.5% reporting reduced movements in PWS infants. This is consistent with the figures of 85% and 88% reported in the studies of Miller et al (23) and Gross et al (9), both of which assessed FMs using antenatal ultrasound.

In our study mothers who had only experienced one pregnancy with a PWS infant were less likely to score FM as low. However, scores given by uniparous mothers were still significantly lower than those for the unaffected babies of other mothers. Furthermore, preterm infants had a lower median score for FMs than term babies, but despite this term babies’ scores were still significantly lower compared with unaffected babies. Table 3 shows that eight of twelve patients diagnosed ≥4 weeks of age had reduced FMs, awareness of which could have secured earlier diagnosis. Subjective maternal reporting, even for uniparous women, appears reliable and increased awareness of this among obstetricians and neonatologists is important (18).

The most constant feature of PWS at birth is hypotonia, Dudley and Muscatelli citing a 97% incidence (17). Gunay-Aygun et al (24) cite “hypotonia with poor suck” as the sole factor from birth to two years, sufficient to prompt genetic testing for PWS.Feeding difficulty is also common in PWS, NGF being instituted in 86% of our cohort. Late-diagnosed infants had shorter durations of NGF reflecting failure to recognise the condition and its severity. In the few cases where NGF was not commenced, some mothers described enormous difficulties in feeding their infants. The value of prompt diagnosis in reducing the period of hospitalisation and duration of NGF has been shown by Bacheré et al (25). The very high rate of male hypogonadism in PWS is confirmed by this study with a prevalence of 90% amongst preterm boys compared with 30% prevalence reported in unaffected preterm boys (26).

During the study period the median age at diagnosis of PWS appeared to improve with a fall from 9 to 5 days between 2000-2009 and 2010-2015. However it is important to note that the number of as yet undiagnosed children with PWS is currently unknown, and that the true median age at diagnosis may prove to be higher.

Consistent with previous studies we have shown an increase in parental age with mUPD15 compared with del15q (16). We also showed that the ratio of mUPD15 to del15q rose during the study, consistent with the rise in parental ages in Scotland between 1977 and 2015-from 26.1 to 29.4 and 28.6 to 32.3 years for mothers and fathers respectively (20). The relationship between maternal age and disomy has been hypothesized to result from a phenomenon known as trisomy rescue (16,27). No other significant difference was found between the two genotypes.

Early diagnosis in PWS is highly desirable. Therapies to counter muscular hypotonia, including GH therapy and physiotherapy training programmes, may be especially useful in the early years. During this period PWS infants and young children may have impaired motor development and skill acquisition which has been reported to affect further development of social abilities and cognitive function (5). GH production is reduced at all ages in PWS but is more marked at younger ages and reduced muscle mass is present from infancy so that early GH therapy may alleviate deficits associated with PWS hypotonia, including improved cognitive development (7,8). Moreover, feeding difficulties are a major problem during infancy and early detection of PWS enables speech and language therapy help with this problem as well as with language acquisition and cognitive development. Indeed, so prevalent are feeding difficulties and the need for NGFs in PWS that this diagnosis can be virtually excluded in older patients with obesity and developmental delay if there is no history of neonatal feeding problems. Beyond infancy parents and carers need to restrict calorie intake in order to prevent the onset of obesity (5,6). Thus an additional benefit of early diagnosis is the opportunity to educate parents and others involved in the welfare of the patient as to the natural history of this complex condition and to not only care for the child, but also to seek help themselves, when necessary (5,7,8).

Given the typical features of PWS, particularly the findings of hypotonia and feeding difficulty at birth, it might be expected that diagnosis would be consistently early. The additional feature of cryptorchidism in almost all males is most helpful, especially when combined with hypotonia. By contrast, hypoplasia of the labia minora was documented in only a minority of female patients. This partly reflects the lack of systematic documentation during the study period, but also the limited diagnostic value of such a subjective feature. The finding of unexplained hypotonia at birth, especially if accompanied by undescended testes in boys, should immediately prompt a search for the phenotypic features of PWS. These include characteristic up-slanting eyes with narrow palpebral fissures (so-called “almond eyes”), narrow nose, bi-frontal narrowing and sticky saliva resulting in the “string sign” (28), small hands and feet, and weak cry. Whilst we decided that it was not possible in this retrospective study to accurately quantify and evaluate these traits individually, a constellation of these features in a hypotonic infant is an important adjunct to diagnosis. However, we would argue that it is actually making the essential connection between neonatal hypotonia and the possibility of PWS in the first place-which only then prompts a search for phenotypic features-which is essential and yet not being made consistently in clinical practice in our experience. Thus our study shows that in Scotland diagnosis of PWS was first suspected well after the first week of life in 34% of patients during the past 15 years. This is despite the presence of hypotonia in all 12 of the late-diagnosed cases with recourse to NGF in nine, reduced FMs in eight, and cryptorchidism in 5/6 boys.

Also, the distress caused to parents when diagnosis is delayed, even for a matter of days, should not be underestimated, especially when investigations for neurological disease, such as MRI and muscle biopsy are contemplated. The mother of patient 7 in Table 3, eventually diagnosed at 9 months, wrote “As the hospital didn’t know what was wrong we were not allowed to go home until our daughter could take the bottle. We secretly cup fed her so as to be able to get home as they wouldn’t let us tube feed. On finding out about her condition we realised that she ticked all the boxes and we feel that the hospital should have recognised the condition. Instead we were told she had cerebral palsy.”

### Study Limitations

The main limitation of our study was its retrospective nature. However, in including all genetically proven PWS patients who have attended our clinic up to 2015, our cohort size was maximised. In addition, a temporal analysis of the speed of clinical and molecular diagnosis at a dedicated, specialised PWS clinic was made possible. A further limitation of our study was the subjective nature of data obtained from the mothers, especially concerning the degree of FM in affected and unaffected children. It is reassuring to compare the subjective recall of our mothers which proved to be consistent with the radiologically measured degree of FM reported by Miller et al (23) and Gross et al (9).

In our opinion the diagnosis of PWS can and should be made within days of birth provided that the hypotonia and the feeding difficulties, which will invariably be evident to the mother and midwife, are appreciated. Subsequent rapid genetic confirmation of PWS is essential since previous work in our centre showed clinical misdiagnosis in 11/31 patients at the time our multi-disciplinary clinic was established (29). In the present study only one patient did not show molecular genetic evidence of PWS despite fulfilling the Holm criteria.

### Conclusion

We believe that, although features such as preterm birth, LBW, SGA, operative delivery and malpresentation are not specific to PWS, their combined presence in the context of hypotonia and feeding difficulties should evoke PWS. Indeed it has been suggested that when a combination of polyhydramnios, SGA and asymmetric intrauterine growth co-exist in the absence of morphological abnormalities, prenatal methylation studies for PWS should be performed (9).

## Figures and Tables

**Table 1:**
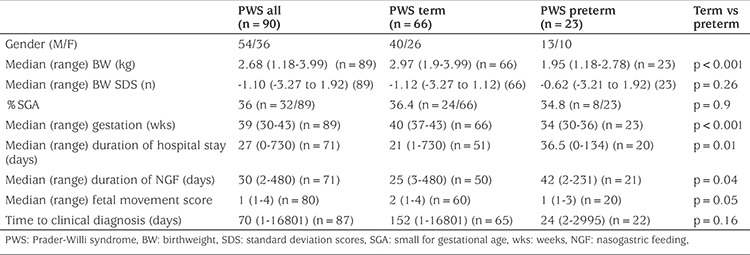
Prenatal, perinatal and postnatal characteristics of 90 patients with Prader-Willi syndrome seen in a single centre between 1991 and 2015 according to term and preterm status

**Table 2:**
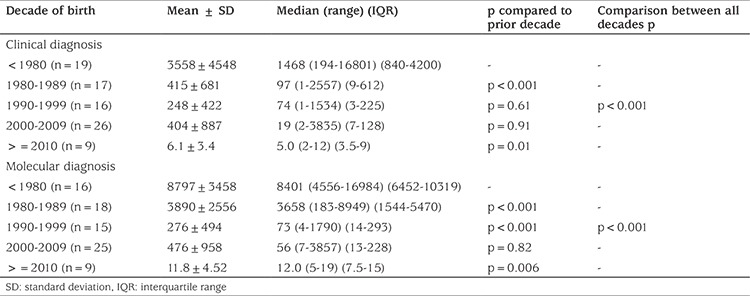
Mean ± standard deviation and median (range) times (days) to clinical (n=87) and molecular (n=83) diagnosis by decade of birth

**Table 3:**
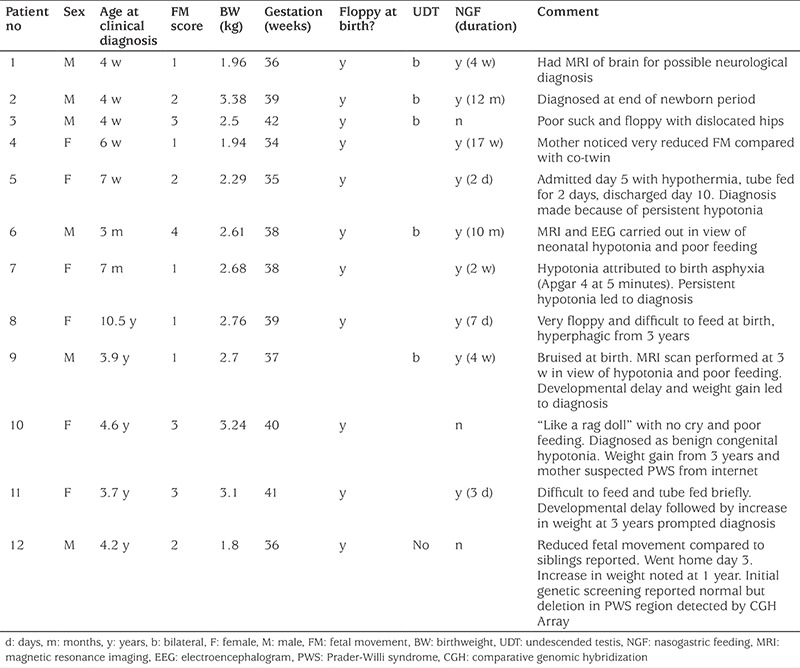
Clinical data on 12 of 35 children with Prader-Willi born from the year 2000 onwards in whom clinical diagnosis was made at or after 28 days

**Table 4:**
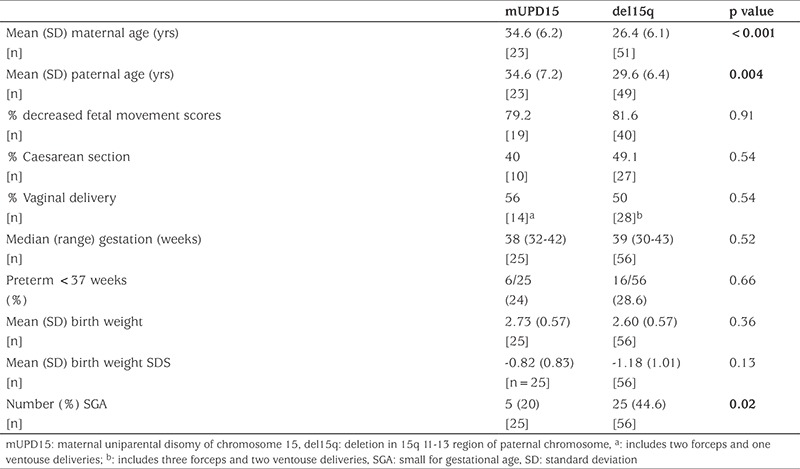
Parental, fetal and perinatal characteristics of 90 patients with Prader-Willi syndrome seen in a single centre 1991-2015 according to molecular genetic defect
